# Influenza Vaccines: Current Status, Adjuvant Strategies, and Efficacy

**DOI:** 10.3390/vaccines13090962

**Published:** 2025-09-11

**Authors:** Vijay Reddy Mokalla, Shirisha Gundarapu, Radhey S. Kaushik, Mrigendra Rajput, Hemachand Tummala

**Affiliations:** 1 Department of Pharmaceutical Sciences, College of Pharmacy & Allied Health Professions, South Dakota State University, Brookings, SD 57007, USA; vijay.mokalla@sdstate.edu; 2 Department of Biology and Microbiology, South Dakota State University, Brookings, SD 57007, USA; shirisha.gundarapu@sdstate.edu (S.G.); radhey.kaushik@sdstate.edu (R.S.K.); 3 Richard A. Gillespie College of Veterinary Medicine, Lincoln Memorial University, Harrogate, TN 37752, USA; mrigendra.rajput@lmunet.edu

**Keywords:** influenza virus, viral pandemics, vaccine, adjuvants, vaccine formulations

## Abstract

The influenza virus is one of the major global health concerns, causing significant morbidity and mortality in both humans and animals, with substantial impacts on public health. Vaccination remains the primary strategy for managing influenza virus infections; however, the virus undergoes frequent genetic changes through antigenic drift and shift. These mutations lead to new seasonal strains that evade pre-existing immunity. These mutations can potentially result in virulent strains that could trigger future pandemics. Therefore, developing a vaccine capable of providing robust protection despite these genetic changes is essential. Vaccine adjuvants are essential for boosting and directing the immune system’s response, broadening the spectrum of protection, and reducing the amount of antigen required to achieve protection, which is particularly valuable in the face of rapidly evolving strains and during pandemics. Recent advances in adjuvant design and formulation strategies have demonstrated promising improvements in both the overall potency and durability of influenza vaccines, importantly, significant reductions in losses due to influenza infection. This review highlights the current status of different types of influenza virus vaccines, their benefits, and challenges. Further, the review focuses on the role of adjuvants, discussing their advantages, limitations, and methodological approaches, while also considering their potential contribution in developing a universal flu vaccine intended to provide extensive and lasting protection.

## 1. Introduction

Influenza virus is a highly contagious pathogen that infects multiple species, including humans, pigs, and birds. It is a negative-sense, segmented RNA virus classified under the family Orthomyxoviridae. The virus is divided into several types: A, B, C, and D based on their genetic makeup, antigenicity, and host spectrum [1]. The genomes of Influenza A and B viruses consist of eight RNA segments, while Influenza C and D viruses have seven segments [2]. Humans, birds, pigs, and horses are among the hosts susceptible to Influenza A virus infection [3,4]. Whereas the primary host for the Influenza B virus is humans, though also identified in seals [5,6]. Influenza C infects primarily humans and pigs and occasionally infects dogs [7]. Influenza virus D was recently identified as a livestock virus primarily affecting cattle with sporadic cases in pigs and goats [8]. Among all influenza viruses, the Influenza A virus is considered the most pathogenic to humans, due to its ability to infect multiple species, undergo rapid genetic changes, and adapt to new hosts [9]. The segmented nature of influenza A genomes permits the mixing of RNA segments from different strains within the same host, creating new viral variants [10]. Such sudden changes (antigenic shift) have resulted in emergent strains against which little population immunity exists, potentially sparking pandemics [11,12]. In addition, continuous antigenic drift (accumulation of point mutations) causes influenza viruses to constantly evolve, making it challenging to forecast which strain will dominate each season [13]. Further, influenza viruses from animal origin remain a major global health threat because they can mutate or reassort to gain efficient human-to-human transmission, potentially sparking a severe pandemic (zoonosis) [14].

Influenza virus infection could cause severe life-threatening complications in children, the elderly, and adults with long-term health complications such as diabetes, asthma, chronic kidney disease, or heart disease [15]. In addition to causing health complications, Influenza outbreaks also add significant financial burdens to society. It is estimated that annual influenza infections add $87.1 billion in cost to the US economy and €6 and €14 billion to the European economy [16,17]. A recent study in 2023, based on approximately 20 million influenza-related claims collected over five years (2015–2019), estimated that annual costs of influenza-related hospitalizations in the United States were about $8.0 billion annually [18].

Vaccination remains a highly effective strategy to prevent influenza infection and to limit its transmission. Based on surveillance of potential circulating influenza viruses, viral strains for the vaccines are selected that are likely to induce a protective immune response against the circulating strains. Influenza vaccines designed based on the surveillance data are typically formulated as either quadrivalent vaccines, containing two influenza A strains and two influenza B strains, or trivalent vaccines, which include two A strains and one B strain [19]. Although this strategy has been effective, influenza viruses have a high mutation rate, particularly due to antigenic drift and occasional antigenic shifts, which complicates accurate prediction of dominant strains. Consequently, seasonal vaccines sometimes fail to completely match the circulating strains, reducing their protective efficacy [20,21]. For these reasons, despite widespread vaccine availability, influenza still causes significant illness and deaths worldwide, and it remains a serious health concern [22]. Further, protection from modern influenza vaccines is predominantly strain-specific, leaving individuals vulnerable to viruses that have drifted or shifted [23].

To address this limitation, the advancement of next-generation influenza vaccines incorporating novel adjuvants can significantly boost both the magnitude and durability of the immune response [24]. Adjuvants play a pivotal role in enhancing vaccine efficacy by activating innate immune pathways, antigen presentation, and promoting long-lasting adaptive immunity. Their significance becomes particularly evident in the context of influenza vaccines, where antigenic drift, strain variability, and reduced responsiveness in vulnerable populations such as the elderly often limit protective efficacy. Several adjuvants have been identified for their potential to elicit cross-protective immunity against multiple influenza strains [25].

The primary objective of this review article provides a foundational overview of the influenza virus and examine conventional influenza vaccines, detailing their mechanisms of action and inherent limitations, particularly their strain-specific protection. In addition, it highlights the critical role of vaccine adjuvants in enhancing the immunogenicity and overall efficacy of current influenza vaccines, as well as their capacity to elicit broad, cross-protective immune responses. Finally, the review explores emerging strategies and innovative approaches aimed at improving influenza vaccine performance and shaping the future landscape of influenza prevention.

## 2. Methods

This review employed a narrative approach, aiming to provide a comprehensive synthesis of current knowledge on the influenza virus, the types of influenza vaccines available, their modes of action, and seasonal influenza vaccines, including trivalent and quadrivalent formulations. The review also focused on vaccine epidemiology, health outcomes, and related policy changes. A narrative review was chosen to allow a broad discussion of scientific, regulatory, and public health perspectives, rather than conducting a quantitative meta-analysis.

Relevant literature was identified through searches of multiple databases and official sources, including Google Scholar, PubMed, U.S. Food and Drug Administration (FDA), World Health Organization, Centers for Disease Control and Prevention (CDC), and the United States Department of Agriculture (USDA). Literature research was conducted from June 2024 to August 2025, without restrictions on publication date, including sources that provided peer-reviewed scientific evidence or information from official governmental and organizational websites. This approach allowed for the integration of emerging trends in influenza vaccine development, while retaining historical discoveries and foundational data essential for a comprehensive review. There was no language restrictions applied; however, only sources accessible in English were included. Inclusion criteria comprised studies, official reports, and guidance documents addressing influenza vaccine composition, effectiveness, cost-effectiveness, epidemiology of circulating strains, and public health recommendations. Exclusion criteria included non-peer-reviewed opinion pieces, unpublished data, and studies unrelated to human influenza vaccine. Being a narrative review, this study has inherent limitations. It does not employ systematic quantitative synthesis or meta-analysis, which may introduce selection bias. The results and conclusions rely on the availability and quality of published information and official reports during the specified timeframe. Nevertheless, the use of multiple reputable databases and regulatory sources provides a comprehensive overview of recent developments in influenza vaccination.

## 3. Influenza Virus Pandemics, Seasonal Outbreaks, and Zoonosis

Influenza viruses have posed a persistent threat to global public health through both sporadic pandemics and recurring seasonal outbreaks. The pandemic H1N1 1918, the H2N2 pandemic 1957, the H3N2 pandemic 1968, and the H1N1pandemic 2009 are the four major influenza pandemics in recent human history caused by the influenza virus A. The 1918 influenza pandemic infected approximately one-third of the global population and caused an estimated 50 million deaths, whereas the H2N2 (1957) and H3N2 (1968) pandemics each resulted in about one million fatalities [26]. The 2009 influenza pandemic resulted in an estimated 151,700 to 575,400 deaths [27]. The 1957 H2N2 pandemic virus contained three gene segments derived from avian influenza A viruses, while the 1968 H3N2 pandemic virus carried two avian-origin gene segments. In contrast, the 2009 H1N1 pandemic virus possessed a unique genomic composition, and it contained gene segments originating from avian, human, and swine influenza A viruses [28]. In addition to these pandemics, scientists have observed seasonal patterns in influenza virus infections, correlated with distinct waves in the northern and southern hemispheres [29]. In seasonal variations, the influenza A virus undergoes frequent mutations, especially in its HA (Hemagglutinin) and NA (Neuraminidase) surface proteins. Influenza A virus has 18 distinct hemagglutinin (HA) subtypes and 11 neuraminidase (NA) subtypes. The combination of different HA and NA subtypes in the virus leads to distinct patterns of infection and severity in humans, resulting in seasonal influenza infections affecting millions of people each year [30]. Among the Influenza A virus subtypes, only H1, H2, H3, H5, and H7 have been shown to infect humans. Although sustained human-to-human transmission is rare, avian influenza A subtypes such as H5N1 and H7N9 have occasionally infected humans and are associated with high mortality rates. The high mutation rate emerging from frequent replication errors in influenza virus genes is attributed to the lack of proofreading activity in its RNA-dependent RNA polymerase [31]. Non-structural genes of influenza A and B viruses have been reported to have annual mutation rates of around 2.0 × 10^3^ and 0.6 × 10^3^ nucleotide substitutions/sites, respectively [32]. Such changes help influenza viruses avoid host immune surveillance and cause seasonal infections. Every winter, multiple seasonal influenza virus strains circulate, causing influenza infection [33,34]. According to estimates from the World Health Organization (WHO), seasonal influenza virus infections result in three to five million cases of serious illnesses, which lead to 290,000-650,00 deaths annually worldwide [35,36]. Influenza has a significant negative impact on human health, particularly in those 65 years of age, and is responsible for 50–70% of flu-related hospitalizations and 70–85% of flu-related deaths, even though the COVID-19 pandemic has caused a decline in influenza activity in recent seasons (2020–2021 and 2021–2022) [37,38].

Influenza A virus infects diverse species, encompassing humans, pigs, and birds, and is considered zoonotic [39]. The likelihood of a large-scale epidemic caused by an emerging influenza A virus increases when reassortment occurs between influenza strains from different species [40]. Pigs are considered important ‘mixing vessels’ because they express both α-2,3-linked and α-2,6-linked sialic acid receptors in their respiratory tract, which allows them to be co-infected with avian and human influenza viruses [41,42]. This dual susceptibility facilitates viral reassortment, increasing the likelihood of generating novel strains with pandemic potential. Migratory waterfowl and shorebirds, on the other hand, act as natural reservoirs of influenza A viruses and contribute to their global spread along migratory flyways [43]. Together, reassortment events in pigs and environmental dissemination by migratory birds drive the continuous evolution, adaptation, and spread of influenza viruses, sustaining their zoonotic threat to human health. In the United States, on 25 March 2024, a multistate outbreak of highly pathogenic avian influenza (HPAI) A(H5N1) in dairy cattle was first reported [44]. This represents the first documented occurrence of avian influenza viruses in U.S. dairy cows. This HPAI H5N1 strain, classified as reassortant genotype B3.13, crossed into dairy cattle, causing unusually high transmission among them [45]. This virus has been reported to spill over into cats and humans. Sporadic human infections have occurred, primarily resulting from close contact with infected dairy cattle, especially through exposure to contaminated milk, while no sustained human-to-human transmission has been observed [45,46]. Recently, several subtypes of avian influenza virus (AIV) have been identified as zoonotic, directly transferring from birds to humans [47]. Among these, recently, human infections with H5N1, H7N9, and H9N2 have risen notably [48]. Current zoonotic H7N9 viruses emerged due to reassortment of viruses from various bird species. While sustained human-to-human transmission has not been documented with H7N9, seroprevalence studies suggest the presence of previously unrecognized asymptomatic infections, warranting more surveillance. This raises concerns about the virus’s potential to adapt for efficient human-to-human transmission, underscoring the risk of a future pandemic and emphasizing the urgent need for effective vaccines to mitigate this threat.

## 4. Animal Model for Influenza Virus Vaccines

Scientists need suitable animal models to test the efficacy of vaccines. An ideal animal model should be susceptible to the influenza virus and exhibit clear signs of infection. Several animals are commonly used for influenza virus studies, including mice, ferrets, guinea pigs, cotton rats, non-human primates, and pigs. Although mice are frequently used animal models in influenza research, most human influenza viruses require adaptation through serial passage to infect laboratory mice, and the clinical course of the disease in mice often does not accurately reflect human disease [49]. Ferrets, on the other hand, are considered a better model for human influenza disease compared to mice. They are similar to humans in sialic acid composition (both linkage types and absence of Neu5Gc), lung structure, susceptibility, and disease progression [50]. However, they could show vaccine-associated enhanced respiratory disease (VAERD), a condition where, instead of protecting against infection, a vaccine leads to worsened respiratory illness when the vaccinated individual is later exposed to the actual virus [51]. Guinea pigs are another animal model; they are susceptible to various influenza virus types, including influenza A (H3N2, H1N1), B, and D [52,53]. Guinea pigs have both SAα2,3-Gal and SAα2,6-Gal receptors in their nasal tract and trachea, similar to pigs and humans. However, they are less commonly used due to cost or other model limitations [54]. Cotton rats serve as a valuable small animal model because human influenza strains can replicate in their lower respiratory tract without prior adaptation, resulting in disease development. Similarly, non-human primates, including rhesus and cynomolgus macaques, can be experimentally infected and may develop illness, offering a model that closely reflects human responses due to their evolutionary proximity. However, their use is limited due to ethical considerations, high cost, and practical challenges [55].

Among all the animal models discussed, pigs represent one of the best options because they share many physiological similarities with humans, particularly in their respiratory systems and immune responses. Further, they are also naturally susceptible to influenza virus infection [56]. Pig respiratory tracts exhibit a receptor distribution (SAα2,3Gal and SAα2,6Gal) comparable to humans, which allows unadapted human influenza A virus strains to infect pigs directly [57]. Furthermore, pigs can mount robust innate, Th-1, Th-2, Th-3 and proinflammatory cytokines, as well as produce influenza virus-specific-IgA antibodies in their lungs, closely mirroring the human immune response [58]. The clinical signs and disease progression of influenza in pigs are also highly comparable to those in humans, reinforcing their value as an outstanding model for studying influenza virus pathogenesis [59].

Beyond studying influenza infections, pigs have also become great models for studying mucosal vaccinations as their nasal structure provides anatomical space and mucosal barriers similar to humans, unlike mouse and ferret models [60]. This has enabled systematic evaluation of intranasal (IN) nanoparticle-based vaccines designed to induce both mucosal and systemic immunity [61]. Initial efforts in this area employed liposome-based formulations, demonstrating that safe and biocompatible lipid carriers can enhance mucosal antigen delivery. For instance, a subunit influenza vaccine incorporating liposomes and the monosodium urate (MSU) adjuvant elicited strong protective immunity in pigs, highlighting the potential of lipid-based carriers to potentiate mucosal vaccine efficacy. These studies laid the groundwork for the development of more versatile nanoparticle platforms [62]. Building on this foundation, researchers explored polymeric nanoparticles, such as PLGA, which offer superior stability, controlled antigen release, and efficient encapsulation compared with liposomes. PLGA-encapsulated inactivated SwIV (Swine influenza virus) H1N2 antigens induced robust T-cell responses and cross-protection against heterologous H1N1 challenge, even when mucosal antibody induction was modest. These findings illustrate the advantages of polymeric nanoparticles in eliciting durable and cross-protective immunity [63]. Recently, attention has shifted to plant-derived nanoparticles, such as Nano11 derived from sweet corn-based Cationic Alpha-D-Glucan Nanoparticles, which combine natural biocompatibility with immunostimulatory properties. Intranasal administration of Nano11–SwIAV (Swine influenza A viruses), with or without Stimulator of interferon genes (STING) agonist ADU-S100, promoted cross-reactive mucosal IgA, systemic IgG, and cytotoxic T lymphocyte responses in pigs challenged with heterologous and pandemic H1N1 viruses. While viral load reduction was moderate, these results demonstrate the promise of plant-based nanoparticles as safe, scalable, and cost-effective alternatives for large-scale mucosal vaccination [64]. Extending these observations, investigations with modified Nano-11 constructs revealed that intranasal delivery of Nano-11-Kag combined with (polyriboinosinic: polyribocytidylic acid (poly(I:C), stimulated markedly higher levels of cross-reactive virus-specific secretory IgA (SIgA) in both the upper and lower respiratory tracts of pigs when compared with a multivalent intramuscular commercial influenza vaccine. Both the Nano-11-KAg-poly(I:C) and a commercial vaccine increased the number of IFNγ-producing T cells. However, adding poly(I:C) to the Nano-11 formulation specifically boosted cytokine gene expression in the draining lymph nodes. The Nano-11-KAg-poly(I:C) also led to high levels of virus-neutralizing antibodies in the bronchoalveolar lavage fluid and helped partially reduce both lung pathology and the viral load after exposure. These results indicate that incorporating poly(I:C) markedly enhances the immune responses elicited by Nano-11-based vaccines, highlighting the potential of plant-derived nanoparticles as versatile and effective platforms for developing broadly protective swine influenza vaccines [65]. Alongside plant-derived nanoparticle platforms such as Nano-11, other nanomaterials are also being explored for mucosal vaccination against Influenza A virus (IAV). The intranasal (IN) mist-delivered chitosan-based IAV nano vaccine demonstrated strong mucoadhesive properties, which enhanced both mucosal and systemic immunity in pigs. Vaccinated animals exhibited augmented T- and B-cell responses across the upper and lower respiratory tracts, tracheobronchial lymph nodes (TBLN), and peripheral blood. This was accompanied by elevated levels of virus-specific secretory IgA, systemic IgG, and robust T cell responses against antigenically diverse IAV strains. Importantly, these immune responses correlated with reduced nasal virus shedding, lower pulmonary viral titers, and attenuated inflammatory changes in lung tissue. These findings suggest that the chitosan-based IAV nano vaccine represents a promising candidate for protecting swine herds against the continual threat posed by rapidly evolving influenza viruses [66]. Another study revealed that vaccinated pigs exhibited complete protection against homologous IAV-S (Influenza A virus of swine) challenge, with only one of twelve animals showing minimal nasal viral RNA excretion. Histopathological evaluation revealed an absence of gross or microscopic pulmonary lesions in immunized animals. The Lipid nanoparticles-DNA (LNP-DNA) vaccine elicits robust protective efficacy and represents a promising platform for the rapid development of IAV-S vaccines [67]. Collectively, these studies trace the evolution of intranasal nanoparticle vaccines in pigs: liposomes established proof-of-concept for mucosal delivery, polymeric nanoparticles enhanced immunogenicity and cross-protection, and plant-derived nanoparticles offer a biocompatible and economical option for translational applications. Together, this body of work underscores the pig model’s critical role in advancing nanoparticle-based intranasal influenza vaccines toward human use [68].

## 5. Approved Influenza Vaccines

Globally, there are numerous approved influenza vaccine formulations produced annually. Each platform has distinct production processes and immunogenic properties. Table 1 provides examples of FDA approved influenza vaccines used in seasonal vaccination. In addition to these seasonal influenza vaccines, several FDA approved influenza vaccines are stockpiled for pandemic preparedness. In general, influenza vaccines are updated each year based on WHO surveillance of circulating strains, typically formulated as a quadrivalent influenza vaccine: containing two Influenza A subtypes (H1N1 and H3N2) and two B lineage (Yamagata and Victoria) strains [69]. The selection of strains is made months in advance, and vaccine effectiveness can be substantially reduced if the virus undergoes unexpected drift or if a mismatched strain predominates [70]. These seasonal influenza vaccinations are typically categorized into three groups: live attenuated vaccines, inactivated vaccines, and recombinant hemagglutinin vaccines. All these formulations can be quadrivalent Influenza Vaccine (QIV), trivalent Influenza Vaccine (TIV), or monovalent Influenza Vaccine [71,72].

### 5.1. Live Attenuated Vaccines

Live attenuated vaccines are developed by reducing the virulence of a pathogen through genetic modification or repeated culturing in non-human cells while preserving its ability to replicate and stimulate an immune response without causing illness. For the influenza virus, several key strategies are employed to create these attenuated viruses. One common approach involves introducing temperature-sensitive and cold-adapted mutations. The virus has been modified to replicate efficiently at the lower temperatures found in the upper respiratory tract. However, it is unable to reproduce at the higher temperatures of the lower respiratory tract, which reduces its ability to cause disease [73,74].

FluMist^®^ vaccine is a good example of this approach, with its core genes originating from master donor viruses adapted to cold temperatures, while its surface glycoproteins (hemagglutinin and neuraminidase) are derived from currently circulating influenza strains [75,76]. The intranasal delivery of FluMist^®^ simulates the natural pathway of influenza infection, allowing viral replication in the cooler upper respiratory tract environment, which generates robust mucosal IgA antibodies and provides protection at the primary site of infection. Mucosal protection offers an advantage over traditional injectable influenza vaccines that primarily induce systemic immunity. FluMist^®^ was approved by the US FDA in 2003; the first intranasal influenza vaccine. The quadrivalent formulation contains attenuated strains of two influenza A lineages (AH1N1, A/H3N2) and two influenza B lineages to provide broad-spectrum protection against circulating seasonal influenza viruses [77,78].

Despite its initial success and the benefits of needle-free administration and robust mucosal immune response, FluMist^®^ faced efficacy concerns, particularly against the influenza A(H1N1) pdm09 strain, which led to its temporary withdrawal from use between 2016 and 2018. The reduced effectiveness was largely attributed to diminished replicative fitness of the live attenuated H1N1 virus in the vaccine, resulting in suboptimal immune protection and decreased overall vaccine performance [79,80].

After reformulating with a new strain and supporting new clinical data, 2018, the use of FluMist was resumed for healthy, non-pregnant individuals between the ages of 2 and 49 who do not have a medical history of pre-existing conditions such as allergies or autoimmune disorders [81].

Currently, three main live attenuated influenza vaccines are in clinical use worldwide, each built on different attenuated backbones: the Ann Arbor (utilized in Fluenz/FluMist^®^ by AstraZeneca), the Leningrad (used in Ultravac by Microgen), and Nasovac (produced by the Serum Institute of India). These vaccines represent important advancements in influenza prevention strategies, particularly for populations who benefit from the mucosal immunity they generate [29,82].

In summary, live attenuated influenza vaccines are designed by reducing viral virulence while preserving the ability to replicate and stimulate immune responses without causing illness. They often incorporate temperature-sensitive and cold-adapted mutations, enabling replication in the cooler upper respiratory tract while limiting growth in the lower respiratory tract, which reduces disease risk. Administered intranasally, live attenuated influenza vaccines stimulate robust mucosal IgA responses at the primary site of infection, alongside systemic immunity. Live attenuated influenza vaccines have shown broad protection against circulating influenza strains, although efficacy can vary with viral replicative fitness and strain composition. A theoretical concern exists regarding reversal of pathogenicity, but no clinically significant cases have been reported in approved vaccines. Safety profiles are generally favorable, with most adverse events being mild and transient, such as nasal congestion, runny nose, or low-grade fever. Pregnant individuals should not receive live attenuated influenza vaccines, as they are contraindicated due to potential risks to the fetus. Serious adverse events remain rare, and live attenuated influenza vaccines provide an important alternative to injectable vaccines, particularly for populations benefiting from needle-free administration and mucosal immunity.

### 5.2. Inactivated Vaccines

Inactivated viral vaccines are produced by rendering viruses non-infectious through chemical or physical treatment, most commonly using agents such as formaldehyde or β-propiolactone, which crosslink viral proteins and nucleic acids to abolish replicative capacity [83]. Based on their manufacturing processes and composition, these vaccines are classified into split virus, whole virus, and subunit vaccines, which were among the first-generation of influenza vaccines developed in the 1940s [72].

Vaccines containing the whole virus are capable of penetrating target cells; however, because the virus has been rendered non-replicative, it fails to multiply and thus cannot stimulate an adequate immune response [84]. While they induce robust immune responses, safety concerns emerged due to diverse reactions and pyrogenicity, largely attributed to the lipid components of viral envelopes [85]. To overcome these issues, split virus vaccines were introduced in the 1960s. These are produced by inactivating the virus and subsequently treating it with ether or detergents (e.g., Triton X-100) to disrupt the viral envelope and remove lipids and glycoproteins [86]. The removal of these components significantly improves vaccine tolerability by reducing pyrogenicity and adverse effects [87].

Although these viruses can no longer replicate or cause disease, they retain enough of their original structure to stimulate the immune system to produce a protective response [88]. Split influenza vaccines are effective in providing immunity to individuals who have already been exposed to the influenza virus. They help boost the immune response by presenting key viral proteins to the immune system when these individuals encounter the virus again. However, these vaccines do not provide enough immunity for newborns or very young infants who have not previously encountered the influenza virus [89]. This is because split vaccines have lower immunogenicity due to a lack of immunogenic components that stimulate the innate immune response, which are removed during the splitting process. These immunogenic components, such as Single-stranded RNA (ssRNA), typically act as Pathogen-Associated Molecular Patterns (PAMPs) and serve as ligands for innate immune receptors, including Toll-like receptors (e.g., TLR7), which activate a strong antiviral immune response. The loss of ssRNA and other viral components in split vaccines makes them less effective as compared to whole-virus or live-attenuated vaccines. Therefore, split vaccines often need adjuvants to stimulate the innate immune system [90,91].

Fluzone^®^ (Sanofi Pasteur) and Fluvirin^®^ (Seqirus) are prominent examples of inactivated split influenza vaccines, offering improved safety profiles compared to whole inactivated virus formulations. For elderly populations (≥65 years), who often exhibit immunosenescence and reduced vaccine responses, higher-dose formulations have been developed. Fluzone High dose^®^ (Sanofi Pasteur), which contains 60 µg HA per strain (Four times the standard dose), has demonstrated superior efficacy in this demographic. A randomized controlled trial with 31,989 participants aged 65 and above found that the high-dose inactivated influenza vaccine reduced influenza cases by 24.2% more than the standard-dose vaccine. Additionally, it offered improved protection against pneumonia associated with influenza (ClinicalTrials.gov ID: NCT01427309) [92,93].

### 5.3. Recombinant Subunit Vaccines

Recombinant subunit vaccines represent a newer approach that avoids the need for propagating live influenza viruses. Subunit vaccines incorporate purified viral proteins or components of individual proteins as antigens, which are often produced by recombinant DNA technology. In influenza vaccines, these proteins or peptides are primarily from the hemagglutinin (HA) and neuraminidase (NA) surface glycoproteins, which are critical for generating protective immune responses while eliminating potentially reactogenic viral components [94]. By eliminating other viral constituents, subunit vaccines tend to be less reactogenic than whole or split-virus vaccines while still stimulating protective antibodies to HA and NA. Although subunit vaccines induce protective immunity with higher safety, they often require immune activators (vaccine adjuvants) to enhance the immune response [85]. Vaccine adjuvants are discussed in detail in Section 8. As an example of a subunit vaccine, the recombinant HA vaccine Flublok^®^ (first licensed in 2013) is produced in insect cell culture using a baculovirus vector and contains three or four HA proteins corresponding to the recommended strains, without any viral nucleic acid or egg proteins [81,95]. Since the flu season of 2020–2021, Flublok^®^ is accessible in the U.S. and a few other EU nations. The Recombinant hemagglutinin vaccine contained 3 times the HA protein content of a standard dose, egg-derived, quadrivalent inactivated influenza vaccine. Flublok^®^ administration was associated with a 30% reduction in influenza-like illness [96,97,98]. Flublok^®^ can be produced faster than egg or cell-based vaccines because it does not rely on chicken eggs or seed viruses, eliminating the need for virus adaptation in these systems [96,99]. Additionally, the use of embryonated chicken eggs for virus cultivation can result in egg protein contamination, potentially increasing the risk of allergic reactions in susceptible individuals.

The WHO annually updates influenza immunogen compositions to reflect antigenic drift in circulating strains, thereby strengthening host immune protection against three or four distinct viral lineages [100]. Most influenza vaccines are trivalent or quadrivalent, combining two A strains (A/H1N1 and A/H3N2) and one or two B strains; their effectiveness fluctuates yearly according to strain match with circulating viruses [101,102].

In summary, each of the above vaccine types has demonstrated varied efficacy in reducing influenza illness and complications in their indicated populations [103]. Inactivated split vaccines are the most widely used due to their balance of immunogenicity and safety. Live attenuated vaccines offer the advantage of mucosal immunity and ease of intranasal administration, though they require careful use in specific age groups. Recombinant vaccines eliminate the need for eggs and can be produced faster, which is valuable for pandemic response. A key point is that vaccine effectiveness (VE) varies annually, depending on multiple factors such as the match between vaccine strains and circulating viruses, population immunity, and vaccine type.

### 5.4. Multivalent Influenza Vaccines

Influenza vaccines contain multiple strains because different types of influenza viruses circulate simultaneously and evolve rapidly through processes such as antigenic drift and, less commonly, antigenic shift. The vaccines (e.g., live attenuated vaccines, inactivated vaccines, and recombinant hemagglutinin vaccines) can be categorized as monovalent, trivalent, or quadrivalent based on the number of virus strains included in the formulation. These strains are selected to address the evolving nature of the influenza virus.

The United States transitioned to quadrivalent influenza vaccines during the 2013–2014 flu season, after observing new influenza B strains circulating in the population. These quadrivalent influenza vaccines, which were implemented in vaccine formulation to address the unpredictability B lineage circulating. Global surveillance has not detected influenza B/Yamagata lineage viruses since March 2020, after the COVID pandemic [104,105]. Updated epidemiological data also showed the absence of a public health risk from B/Yamagata viruses since 2016, much before the COVID pandemic [106]. Consequently, both the WHO and the FDA’s Vaccines and Related Biological Products Advisory Committee (VRBPAC) have recommended eliminating the B/Yamagata component from influenza vaccines. Similar recommendations were implemented by the European Medicines Agency (EMA) and Emergency Task Force (ETF) as well [107]. Therefore, trivalent vaccines include the same two influenza A strains (H1N1 and H3N2) but only one influenza B lineage (Victoria) was introduced [108]. Trivalent vaccines are projected to save $100 million per year in direct medical costs. When considering other indirect costs, total savings could reach $7.1 billion per year, including savings related to including one more strain in vaccine formulation, materials, and supply chain expenses [19,109,110,111,112]. The transition to trivalent influenza vaccine is occurring in at least 40 other countries, in addition to the USA, as of May 2025. The 2025–2026 egg-based trivalent vaccines are formulated with A/Victoria/4897/2022 (H1N1) pdm09-like, A/Croatia/10136RV/2023 (H3N2)-like, and B/Austria/1359417/2021 (B/Victoria lineage)-like viruses [113].

## 6. Efficacy of Licensed Vaccines

The annual effectiveness of influenza vaccines is evaluated by public health organizations, such as the CDC has monitored seasonal influenza vaccine effectiveness (VE) each year since 2004, except in 2020–2021 when influenza activity was extremely low due to COVID-19 mitigation measures [114]. VE is typically measured by observational studies (test-negative design) and indicates the percentage reduction in influenza illness among vaccinated individuals compared to unvaccinated. Vaccine efficacy/effectiveness can vary widely by season, subtype, age group, and vaccine type. As noted, a key determinant is the antigenic match between vaccine strains and circulating strains [115,116]. Even if the vaccine strains are well-matched, moderate efficacy (40–60%) is common because of other factors, such as host immune history. In mismatch years, efficacy can be low, especially for rapidly drifting viruses, such as A/H3N2 viruses. For example, during the 2021–2022 influenza season in Europe, vaccine effectiveness (VE) against A/H1N1 was 79%, compared to 36% against A/H3N2 across all age classes. These estimations were calculated after eliminating people who tested positive for SARS-CoV-2 from the control group [117]. The lower VE of H3N2 vaccines were attributed to the antigenic drift observed in the seasonal virus [118]. To improve vaccine efficacy for the H3N2 strain, some manufacturers have modified the virus by passaging it through chicken eggs. However, because H3N2 viruses replicate poorly in eggs, egg adaptation often results in mutations, particularly in the hemagglutinin (HA) protein, which can alter the virus’s antigenic properties. These mutations, including those at key residues such as those located at positions 160, 186, and 225 of HA, can impair the immune response and reduce vaccine effectiveness [119,120]. Additionally, egg adaptation preferences vary by viral clade, and some strains exhibit incompatibilities between egg-adaptive mutations and natural amino acid variants. For example, mutations like L194P, which are commonly associated with egg adaptation, may not be compatible with more recent human H3N2 strains, which have evolved to favor different mutations [121,122]. These incompatibilities can affect virus fitness and the virus’s ability to produce effective immune responses in vaccine formulations [123]. Although influenza vaccine effectiveness (VE) has shown overall improvement, particularly against H1N1 strains, no consistent enhancement has been demonstrated for H3N2 strains [124]. Despite overall improvement in the VE for influenza vaccines, especially with H1N1 strains, no such clear trend was evident for H3N2 strains. It is important to note that influenza vaccine efficacy (VE) is generally highest in healthy younger adults, but significantly lower in vulnerable groups such as young children (under 2 years of age) and older adults, due to immunosenescence [125]. To improve vaccine effectiveness in these populations, newer influenza vaccines incorporate strategies such as high-dose formulations (e.g., Fluzone High-Dose^®^) and adjuvanted options like FLUAD^®^ and other MF59-adjuvanted vaccines [126]. These vaccines have demonstrated higher VE compared to traditional formulations, indicating the potential benefits of updated vaccine strategies for at-risk populations. Over the past decade, large meta-analyses and multicenter studies have refined our understanding of influenza VE. A comprehensive analysis of U.S. data from 2004–2015 estimated that vaccination prevented millions of influenza cases and tens of thousands of hospitalizations annually, despite an average protective efficacy of approximately 40%. The interim report from six European countries for 2021–2022 found the adjusted VE against A(H3N2) was only ~14% in adults (not statistically significant compared to the non-vaccinated group) due to an antigenic mismatch, whereas a case–control study conducted in the 2009–2010 influenza season reported a vaccine effectiveness [114]. Preventing emergency department visits from influenza A (H1N1) among medical staff at São João Hospital in Porto, the VE was 90.5% (95% CI: 73.5–97.3%). Another study from the same season, involving a cohort of Japanese healthcare workers, demonstrated a VE of 70.5% reducing hospitalizations caused by influenza A (H1N1) [127,128]. Seasonal influenza vaccines have also been shown to lessen the severity of illness in breakthrough cases, with vaccinated individuals having reduced rates of intensive care unit (ICU) admission [117,128,129].

This has driven the development of enhanced vaccines for the elderly (as discussed) and the consideration of a two-dose primary series for immunologically naive children under 9 years of age. Additionally, repeated annual administration has raised concerns about immune interference, as some studies reported that exposure in the prior season may reduce protective efficacy in the current season under certain conditions. Additionally, repeated annual vaccination has raised concerns about interference, as some studies observed that receiving the vaccine prior season may reduce VE in the current season under certain conditions. However, other studies suggest that during seasons with significant antigenic drift, individuals vaccinated in consecutive years may retain better residual protection against drifted strains compared to those who received the vaccine only in the current season. Overall, the consensus is that annual influenza prophylaxis is beneficial, particularly for high-risk populations, even with partial effectiveness. Ongoing efforts aim to optimize formulations and develop next-generation strategies to achieve broader and longer-lasting protection. Overall, the consensus is that annual influenza vaccination is beneficial, particularly for susceptible populations, even if effectiveness is partial. Continued efforts are focused on improving vaccine formulations and developing next-generation vaccines to achieve broader and longer-lasting protection.

## 7. Production Time

The production timeline for seasonal influenza vaccines differs substantially across platforms. Egg-based vaccines, which remain the most widely used, generally require 6–8 months from strain selection to large-scale availability. This extended timeframe is primarily due to the need for viral adaptation and propagation in embryonated chicken eggs, a process that is inherently constrained by biological variability and logistical limitations [130]. Compared to the prolonged timeline of egg-based manufacturing, cell-based vaccines (e.g., using MDCK or Vero cell lines) can be produced more rapidly since pre-established cell banks are readily available, eliminating the need for egg adaptation. While overall manufacturing, including downstream processing and quality control, still requires several months, the upstream production phase is notably shorter. For instance, Flucelvax, a licensed cell-based influenza vaccine, demonstrated that the viral growth stage could be completed within 65–75 h, representing a significant acceleration relative to the egg-based approach [131]. Building on this trend toward accelerated production, recombinant protein influenza vaccines (e.g., Flublok can be produced in ~6–8 weeks) express hemagglutinin directly in insect or mammalian cells, avoiding whole-virus propagation and enabling even greater flexibility in responding to emerging seasonal strains. Collectively, these advances illustrate a clear evolution from slower, egg-dependent methods toward highly flexible, rapid-response platforms, which hold considerable promise for improving both seasonal influenza vaccine availability and pandemic preparedness [132]. This accelerated model is highly promising for future influenza vaccine timelines, especially during pandemics, although regulatory approvals and scale-up logistics remain key limiting factors.

## 8. Limitations of Influenza Vaccines

Current influenza vaccines, while lowering the seasonal influenza-related morbidity and death, possess several critical limitations that undermine their consistent and long-term effectiveness. These vaccines rely heavily on the prediction of dominant circulating strains six to nine months before flu season, often leading to a mismatch between the actual strain and the vaccine due to antigenic shift and drift [133,134]. Further, most of the influenza vaccines in the market primarily target the viral surface protein hemagglutinin (HA), which mutates rapidly. This narrow immunological target makes the flu vaccines less effective when faced with variant or novel strains. This strain-specific, narrowly focused immunity necessitates yearly administration and provides limited protection against heterologous influenza strains. This narrow-focused, strain-specific immunity necessitates annual revaccination and offers limited protection against heterologous strains [135,136]. As a result, vaccine efficacy fluctuates annually, often ranging between 10 and 60% depending on the season and past exposure. For example, during the 2014–2015 influenza season, significant antigenic drift occurred in the circulating H3N2 viruses. Consequently, the vaccine was only 6% effective against the drifted H3N2 strain, with an overall efficacy of only 19%. Moreover, certain demographic groups, such as older adults, infants, and immunocompromised individuals, exhibit reduced immune response to vaccines, further reducing population-level protection. Technical constraints in manufacturing also contribute to efficacy issues. For example, egg-based vaccine production can introduce mutations that alter viral antigens, making the final vaccine less immunologically matching to the intended strains [119,137,138]. These challenges highlight the urgent need for next-generation vaccines that provide broader, long-lasting, and universal protection.

## 9. Vaccine Adjuvants

Adjuvants are substances added to the vaccine formulations or administered with the antigen to enhance the specific immune response, particularly in inactivated or subunit preparations. They are added to vaccinations to boost their efficacy, particularly inactivated or subunit vaccines. In the context of the influenza vaccine, adjuvants also play a vital role in increasing vaccine efficacy by enhancing immune responses against both matched and antigenically drifted viruses, thus minimizing the effects of antigenic mismatch [81]. By enhancing the magnitude and durability of immune response, adjuvants can reduce the antigen dose (dose-sparing) to elicit a protective immune response and improve the protection in populations with weak immune response, which includes the elderly and infants. These effects are achieved through various mechanisms, including modulation of innate immune pathways and the creation of a localized antigen depot at the injection site [139].

Mechanisms of action of adjuvants:
Adjuvants initiate and potentiate immune responses via multiple pathways:
Adjuvants enhance the immunogenicity of vaccines through a range of complementary mechanisms that activate innate immunity, enhance antigen presentation, and shape adaptive immune responses. Through these multiple mechanisms, adjuvants improve the vaccine immune response through higher antibody titers, broader immune repertoire, and more durable protection compared to non-adjuvanted vaccines [140,141].

1. Activation of Innate Immune Sensors: Toll-like receptors (TLRs) and inflammasomes are two examples of pattern recognition receptors (PRRs) that are activated by many adjuvants. This activation triggers intracellular signaling pathways, including NF-κB and MAPKs, which in turn promote the production of chemokines and proinflammatory cytokines. These molecules create a local inflammatory milieu that recruits immune cells to the injection site and drains into the lymph node [142].
2. Modulation of Antigen Presentation and Uptake: Adjuvants enhance the uptake of co-delivered antigens by antigen-presenting cells (APCs), including dendritic cells (DCs) and macrophages, promoting APC maturation and upregulation of major histocompatibility complex (MHC) molecules, which leads to improved antigen processing and T cell activation [143].
3. Depot Effect and Antigen Retention: A subset of adjuvants, particularly alum and oil-in-water emulsions, generate a localized depot upon injection. The depot effect slows antigen release and maintains antigen exposure in the presence of immune-stimulating signals for a long time, thereby prolonging immune system engagement [140].
4. Cytokine Induction: By stimulating innate immune pathways, adjuvants induce the secretion of cytokines such as IL-6, IFN-γ, and CCL2. These cytokines support the development of effective B and T cell responses, promoting both humoral and cellular immunity [144].
5. Modulation of T-helper cell responses: Different adjuvants can direct the immune response toward either Th1 or Th2 phenotypes. Th1-biased adjuvants enhance cytotoxic T lymphocyte activity and promote IgG2a/IgG3 production, whereas Th2-type adjuvants favor IgG1/IgE production and help T cell differentiation. Some modern adjuvants are designed to promote a balanced Th1/Th2 response, which is especially beneficial for antiviral immunity [145].
6. Inflammasome Activation: Particulate adjuvants such as alum can activate the NLRP3 inflammasome in APCs. This triggers the production of IL-1β and IL-18, potent inflammatory cytokines that further enhance adaptive immune responses [140,141].

In 1926, aluminum salts, specifically potassium aluminum sulfate (alum), were introduced as a vaccine adjuvant that enhanced the immunogenicity of diphtheria toxoid [29,146,147]. Although alum’s success spurred further investigation into immune potentiators, for much of the 20th century, Alum remained as the only adjuvant used for human use. It was not until the late 1990s and early 2000s that newer adjuvants like MF59, AS01, AS03, AS04, AF03, and virosome were licensed, and several of them have been introduced into influenza vaccines [148]. These modern adjuvants offered novel mechanisms of action and greater immunological precision, marking a shift from empirical discovery (alum) to rational design in adjuvant development.

### 9.1. Alum

Aluminum salts such as aluminum hydroxide, aluminum phosphate, and aluminum potassium sulfate (collectively known as alum) have been employed as vaccine adjuvants for over 70 years [149,150,151]. Alum as an adjuvant enhance immune responses through two distinct mechanisms: (i) by increasing the immunogenicity of antigens by forming insoluble aggregates that adsorb vaccine antigens rendering them multivalent and enabling antigen-presenting cells to bind and uptake them [152]; (ii) by creating an inflammatory environment in the injection area through the activation of proinflammatory cytokines, which are released via the NOD-like receptor protein 3 (NLRP3)-inflammasome to promote the maturation of dendritic cells and the activation of T-helper cells, which ultimately enhance the adaptive immune response against the co-injected antigen [148,149,153]. Currently, several licensed human vaccines use alum as an adjuvant due to their well-established safety profile and ability to enhance antibody-mediated (humoral) immune responses. The examples include DPT (diphtheria, pertussis, and tetanus), Hepatitis B (Energix-B^®^, Recombivax HB^®^), Anthrax (BioThrax^®^), and Haemophilus influenzae type b (PedvaxHIB^®^). Based on its success in a myriad of vaccines, several studies have explored alum’s utility in influenza vaccines. Influenza vaccine with alum produced suboptimal enhancement of immune responses. Alum also tends to encourage the Th2 type immune responses, which are categorized by cytokines, such as IL-4 and IL-5, which are more focused on antibody production rather than the cellular immunity needed for long-lasting and broader protection. This prompted the test of other modern adjuvants that produce both cell-mediated and humoral responses [154,155]. However, innovative combinations such as poly-γ-glutamic acid-alum (PGA/Alum) have achieved significantly enhanced humoral and cellular responses against pH1N1 vaccines in mice, including increased IFN-γ CD8+ T-cell frequencies and improved virus clearance [156]. Even early-phase clinical trials on alum-adjuvanted whole-virion H7N9 inactivated vaccines (15 µg HA + aluminum hydroxide) demonstrated acceptable safety but only modest immunogenicity, insufficient to elicit high seroprotection in healthy adults [157]. A review and meta-analysis of prepandemic influenza vaccinations using alum adjuvants found that alum generally induced weaker seroprotection compared to non-adjuvanted formulations [158]. Despite limited success with alum in influenza vaccines, its ability to enhance antibody responses without major safety concerns keeps it relevant. But for influenza, due to antigenic drift and the need for durable, cross-protective immunity, including cell-mediated immunity. Therefore, modern and more potent adjuvants that generate both humoral and cell-mediated immunity, like MF59 and AS03, have taken precedence.

In summary, while alum provides a safe, inexpensive adjuvant for influenza vaccines, its limited ability to generate robust cellular immunity limits its effectiveness. Future strategies focus on combining alum with other immunostimulants to achieve broader and more durable protection [159].

### 9.2. MF59

Oil-in-water emulsion adjuvants have emerged as key components in enhancing the immunogenicity of influenza vaccines, especially during pandemic outbreaks. MF59, an oil-in-water emulsion-based adjuvant developed by Novartis (now CSL Seqirus) composed of squalene stabilized with surfactants, such as sorbitan trioleate and polysorbate 80. It was first licensed in 1997 for use in the seasonal influenza vaccine FLUAD^®^, initially approved in Italy and later authorized in Europe and the U.S for adults aged 65 and older. MF59 does not function via depot formation like alum, as the emulsion is rapidly cleared from the injection site [155,160]. Instead, it enhances immune responses by rapidly recruiting innate immune cells to the injection site. This recruitment stimulates the release of chemokines and cytokines, including CCL2, CCL3, IL-5, IL-8, and IFN-γ, which in turn facilitate robust Th1 and Th2 responses. Further, MF59 increases the phagocytic activity of macrophages and DCs, thereby facilitating better antigen presentation. Antigen-presenting cells migrate from peripheral tissues to lymph nodes, where they activate T and B cells. The emulsion also increases the membrane fluidity of APCs, which may help facilitate endocytosis of vaccine antigens [160,161]. Through multiple mechanisms, MF59 supports a balanced Th1/Th2 profile, making it particularly useful for elderly individuals with waning cellular immunity. Studies examining MF59 in pre-pandemic influenza vaccines, such as H5N1 and H1N1, have highlighted its role in broadening the antibody repertoire. It has been demonstrated that sera from recipients of MF59-adjuvanted H5N1 vaccines showed high levels of antibodies targeting both hemagglutinin (HA) and neuraminidase (NA) proteins. Using phage display libraries, they observed that these antibodies recognized conformational epitopes. The epitopes identified by broadly neutralizing antibodies which are often located in the HA1 subunit, particularly close to the receptor-binding domain (RBD) [162]. Conversely, alum-adjuvanted or non-adjuvanted antigens primarily generated responses against shorter, linear HA1 epitopes and elicited lower frequencies of anti-HA1 and anti-NA antibodies, highlighting limited immune breadth. [163]. Further research on the 2009 pandemic H1N1 influenza vaccine found that MF59 enhanced both the breadth and affinity of antibody responses. Notably, MF59 favored the expansion of antibodies targeting the HA1 globular head domain, critical for neutralizing activity, over the more conserved HA2 stalk region [126]. These findings suggest that MF59 improves not only the quantity but also the quality of humoral responses, enabling better cross-protection against drifted or heterologous viral strains.

MF59-adjuvanted influenza vaccines have shown a robust safety profile across various age groups. Although local and systemic reactions are more frequent than with non-adjuvanted vaccines, they are generally mild to moderate. Injection-site pain is the most common local reaction, while systemic symptoms such as myalgia and headache occur more frequently but resolve without medical intervention. Serious adverse events have not been significantly associated with MF59 use [164,165].

The MF59-adjuvanted trivalent influenza vaccine (aTIV) is shown to be effective in adults aged 65 years and older, providing greater protection compared with QIV and TIV. The aTIV, significantly reduced influenza-related medical encounters, including both illness episodes and hospitalizations, and its performance was comparable to that of high-dose TIV. However, considerable heterogeneity was observed across studies, likely influenced by seasonal variation, population differences, and diverse outcome measures. These results highlight the importance of additional studies to further confirm the relative vaccine effectiveness (VE) of aTIV in different patient groups [166]. Building on these observations, further comparative work reported similar effectiveness of aTIV and high-dose TIV among older adults without underlying risk factors, but higher protection with aTIV in individuals with one or more comorbidities. In particular, aTIV, more effective in preventing influenza-related medical encounters (IRMEs), including outpatient visits and hospitalizations due to influenza or pneumonia. During the 2019–2020 influenza season, this difference became even more evident in patients with cumulative risk factors, whereas no significant advantage was observed in those without comorbidities. Taken together, these findings suggest that while aTIV and HD-TIV may perform similarly in healthier populations and aTIV provides additional benefits for high-risk groups [167]. Adding further weight to these clinical findings, a meta-analysis demonstrated that MF59-adjuvanted vaccines elicited stronger immune responses compared to non-adjuvanted formulations in both healthy adults and the elderly. The improvement was particularly striking in healthy adults, where the relative risk of enhanced immune response was more than twofold, though it remained significant in older populations as well. These results reinforce the evidence that MF59 not only contributes to improved clinical outcomes but also enhances vaccine-induced immunogenicity. Moreover, the analysis proposed that MF59-adjuvanted formulations could be especially valuable in pre-pandemic settings, although additional randomized trials are needed to refine dose optimization and vaccination schedules [168]. Consistent with these immunogenicity improvements, additional research showed that aTIV/aQIV (adjuvanted quadrivalent influenza vaccine) generated stronger antibody responses against heterologous A (H3N2) strains, achieving a 10.7% higher seroconversion rate compared with non-adjuvanted vaccines. Although long-term data on antibody persistence and protective efficacy remain limited, these results align with earlier evidence of the broader immunological benefits of MF59-adjuvanted formulations. Safety evaluations were also encouraging, while mild local and systemic reactions were more frequent in the adjuvanted groups, serious adverse events were rare and not significantly different from non-adjuvanted comparators. Collectively, the accumulated evidence underscores that MF59-adjuvanted influenza vaccines are effective, immunogenic, and generally well tolerated, particularly in older adults and those with elevated risk profiles [169]. MF59 has emerged as a benchmark adjuvant for influenza vaccines, particularly in the elderly, by enhancing both the magnitude and breadth of immune responses. However, continued efforts to improve influenza vaccine efficacy, especially for pandemic preparedness, and designing of universal influenza vaccine, are exploring MF59 in combination with other adjuvants (e.g., TLR agonists) or in next-generation platforms such as mRNA-based vaccines.

### 9.3. AS03

AS03 is another squalene-based oil-in-water emulsion adjuvant, GlaxoSmithKline (GSK). It gained prominence during the 2009 H1N1 pandemic, as it was used in the European pandemic vaccine (Pandemrix^®^) and later incorporated into pre-pandemic H5N1 stockpiled vaccines. AS03 consists of three components: Squalene oil, DL-α-Tocopherol (Vitamin-E), and Polysorbate 80. The presence of tocopherol distinguishes AS03 from MF59 and is considered to enhance its immunostimulatory activity. The presence of tocopherol differentiates AS03 from MF59 and is thought to enhance the immune stimulating properties [170]. Immunologically, AS03 triggers a transient innate immune activation similar to MF59. It has been shown to activate NF-κB-dependent pathways, leading to a local increase in chemokines and cytokines at the injection site and in draining lymph nodes, such as CCL2, CCL3, IL-6, CSF3, and CXCL1, which trigger immune cell movement [171]. AS03-adjuvanted influenza-A (H1N1) pdm09 vaccine elicited higher hemagglutinin antibody titers in adults and especially in the elderly, compared to non-adjuvanted equivalents [172,173]. Although AS03 promotes the expansion of T cells with greater antibody titers, these cells are usually mixed or show a bias for Th2 responses [174,175]. It is also known that the AS03 adjuvant leads to the development of both IgG1 and IgG2a antibodies, which are separate isotypes [176,177]. The mapped antibody-binding regions of immunological serum using whole genome fragment phage display libraries [178]. The AS03-adjuvanted H5N1 vaccination, which targets the conserved H1/H5 area, increased anti-HA2 antibodies tenfold while also expanding antibody repertoires targeting the HA1 domain, notably those targeting the extended HA1 epitopes covering the RBD. AS03 adjuvant was employed, the bound clones’ HA1 to HA2 ratio increased sevenfold, indicating formation of anti-HA1 antibody responses, and the AS03-adjuvanted H5N1 formulation also triggered anti-NA antibodies targeting the C-terminal region adjacent to the sialic acid–binding site [178]. Antibody affinity for appropriately folded HA1, but not HA2, was improved by the AS03-adjuvanted H5N1 vaccine. In adults aged 18–60, the clade 1 (A/Vietnam/1194/2004) AS03-adjuvanted vaccine generated more long-lived memory B cells and cross-reactive polyfunctional CD4+ T cells than the non-adjuvanted formulation [179].

Additionally, cross-reactive and poly-functional CD4+ T cells may aid in the development of broadly cross-protective antibodies and expanded antibody repertoires after the AS03-adjuvanted H5N1 vaccination [180,181]. Human participants responded effectively to AS03-adjuvanted H5N1 vaccinations, and there were no major side effects [172,182,183]. Myalgia and exhaustion were the most frequent systemic adverse responses, while injection-site discomfort was the most frequent local reaction. The majority of the systemic and local adverse events were mild to moderate, subsided on their own in a matter of days, and did not require medical attention [182]. Additionally, it is noteworthy that while the non-adjuvanted vaccine did not increase the number of narcolepsy cases in the United States, adolescents in Sweden and Finland experienced an increase in narcolepsy cases three to six months after receiving the AS03-adjuvanted influenza pandemic 2009 H1N1 vaccine (Pandemrix) [184]. Given that the loss of hypocretin neurons is the pathologic characteristic of narcolepsy, immunological reactions against hypocretin may be the cause of the rise in narcolepsy cases after receiving the Pandemrix vaccine. The influenza pandemic 2009 H1N1 vaccination, which is adjuvanted with squalene emulsion, and the seasonal influenza vaccine, which is not adjuvanted with MF59, did not result in a rise in narcolepsy patients [29,185,186]. The increased incidence of narcolepsy observed following Pandemrix vaccination was likely due to the specific combination of the pandemic H1N1 antigen and the AS03 adjuvant.

### 9.4. AF03

Among these, AF03 is an oil-in-water emulsion-based adjuvant developed by Sanofi Pasteur. AF03 contains squalene, polysorbate 80, and a citrate buffer serving as a stabilizing agent, in addition to cetostearyl ethers and mannitol [187,188]. AF03 was explored during H5N1 vaccine development, resulting in improved immune responses in preclinical models. These promising results facilitated its transition into clinical evaluation as a key adjuvant component of Humenza, Sanofi Pasteur’s split-virion vaccine formulated in response to the 2009 H1N1 influenza pandemic [189]. Although AF03 has been shown to enhance both humoral and cellular immune responses, such as increased production of IL-5 and IFN-γ in animal models, the precise mechanisms underlying these effects remain largely undefined [81]. The adjuvant effect is further substantiated in the influenza-A (H1N1) pdm09 vaccine formulation, which induced robust seroconversion in both primed and unprimed mice. These findings underscore AF03’s capacity to enhance immunogenicity across heterogeneous immune backgrounds, despite the incomplete characterization of its mechanistic pathways [190].

### 9.5. Virosomes

Modern vaccines increasingly prioritize safety by utilizing well-characterized antigens. However, these purified antigens are often small and, consequently, may not elicit a strong enough immune response on their own, and the co-administration of a suitable adjuvant becomes crucial to enhance their immunogenicity. Virosomes act as an adjuvant system, offering the advantages of being biodegradable, non-toxic, and non-immunogenic (they do not trigger an antibody response against themselves). Virosomes are reconstituted viral envelopes classified as virus-like particles (VLPs). They are engineered by integrating viral membrane glycoproteins, such as influenza hemagglutinin (HA) and neuraminidase (NA), into phosphatidylcholine-based unilamellar liposomes, yielding nanoscale vesicles with an average diameter of ~150 nm. Virosomes are reconstituted viral envelopes. They are virus-like particles (VLPs) that incorporate viral membrane proteins (such as influenza HA and NA) into phosphatidylcholine bilayer liposomes, forming unilamellar virosomes with an average size of approximately 150 nm in diameter. Virosomes enhance the immunogenicity of encapsulated antigens without the requirement for conventional adjuvants. One licensed product was Inflexal^®^ V (Crucell/Berna Biotech), a virosomal influenza vaccine used in Europe for many years. The particulate nature of virosomes, along with the hemagglutinin protein’s ability to bind cellular receptors, mediate pH-dependent endosomal fusion, and activate the immune system, contributes to their adjuvant effect. This virosomal adjuvant system enables antigen presentation via both MHC class I and MHC class II pathways, thereby eliciting the robust T-cell and B-cell immune response [191,192]. Following regulatory approval in Switzerland in 1997, the vaccine was subsequently introduced to markets worldwide, marking its global availability. Despite their current unavailability, virosome and virus-like particle (VLP) platforms represent promising strategies for next-generation “universal” influenza vaccines by facilitating presentation of conserved viral antigens to the immune system [81].

### 9.6. CAF01

The development of liposome-based adjuvants has gained attention for their ability to enhance antigen delivery and modulate immune responses. CAF01, a well-characterized cationic liposomal adjuvant, consists of Trehalose 6,6-dibeheneate (TDB) with the cationic lipid dimethyl dioctadecyl ammonium bromide (DDA), a synthetic glycolipid analog of the mycobacterial cord factor, functioning as an immunomodulator [193]. DDA is a positively charged quaternary ammonium compound with two hydrophobic 18-carbon alkyl chains and a charged hydrophilic head group, enabling its self-assembly into liposomal structures that facilitate antigen encapsulation and delivery [194]. The CAF01 significantly amplified both cellular and humoral immune responses induced by TIV in BALB/c mice. Notably, while TIV alone conferred partial protection against heterologous H1N1 virus challenge, the CAF01-adjuvanted TIV (aTIV) provided complete protection, highlighting the adjuvant’s potential in enhancing cross-protective immunity [195]. Similarly, CAF01 significantly enhances the hemagglutination inhibition (HI) titers induced by TIV in ferrets against homologous H1N1 strains; however, this effect was not observed against heterologous virus challenges, indicating a strain-specific adjuvant effect [196,197]. CAF01, while still under preclinical evaluation, has consistently enhanced the cell-mediated and humoral responses in the preclinical influenza model, highlighting its CAF01 potential as a versatile adjuvant platform for future vaccine development.

### 9.7. TLR4 Agonists

The use of immunostimulatory molecules targeting innate immune receptors has become a central strategy in modern vaccine adjuvant design. Among these, Toll-like receptor 4 (TLR4) agonists have gained prominence for their ability to activate dendritic cells and enhance antigen-specific adaptive immune responses. Building on their ability to engage innate immunity, TLR4 agonists activate multiple antigen-presenting cells through signaling pathways that enhance costimulatory molecule expression and cytokine secretion. This promotes dendritic cell maturation and migration to lymph nodes, facilitating effective T cell priming. By inducing balanced Th1/Th2 responses, these adjuvants stimulate robust humoral and cellular immunity. Collectively, these mechanisms underpin durable and cross-protective immune memory against influenza viruses. Research indicates that adjuvants incorporating TLR4 agonists can enhance influenza vaccines by inducing cross-protective immunity. A detoxified form of lipopolysaccharide (LPS), monophosphoryl lipid A (MPL), is produced from the Re595 strain of Salmonella minnesota and serves as a TLR4 agonist and has been incorporated into several licensed adjuvant systems, notably AS01 and AS04, where it contributes to both humoral and cellular immune activation with a well-established safety profile [198,199]. Upon activation, MPL engages TLR4-mediated signaling pathways that enhance vaccine efficacy primarily through immunomodulation of innate immune cells. Specifically, MPL promotes the maturation and migration of antigen-presenting cells (APCs) to lymphoid tissues, where activated dendritic cells upregulate costimulatory molecules, including B7.1 (CD80), B7.2 (CD86), and CD40. These cells also secrete a range of cytokines: IL-6, TNF-α, IL-1β, IFN-β, and IL-12 that are critical for the activation, differentiation, and polarization of B and T lymphocytes. Through this coordinated activation of innate and adaptive immune pathways, MPL enhances both humoral and cellular responses, as demonstrated across multiple species in preclinical and clinical immunogenicity studies [200]. Recent studies have demonstrated that combining monophosphoryl lipid A (MPL), a TLR4 ligand, with poly I:C, a TLR3 agonist, enhances the adjuvant effect when formulating an inactivated A/Puerto Rico/8/1934 (A/PR8) H1N1 influenza vaccine. This dual-adjuvant system substantially increased antigen-specific antibody production, simultaneously attenuating local proinflammatory cytokine secretion and immune cell infiltration. Furthermore, the co-administered adjuvants fostered the development of durable memory B and T lymphocyte populations in murine models, indicative of sustained adaptive immunity. These findings demonstrate the potential of MPL and poly I:C as a complementary adjuvant combination capable of improving both tolerability and immunogenicity of influenza vaccines [201].

In addition to MPL, other synthetic TLR4 agonists have been explored for their adjuvant potential in enhancing influenza vaccine efficacy. Among these, glucopyranosyl lipid adjuvant (GLA) has been formulated primarily in two forms: the aqueous suspension GLA-AF and the stable oil-in-water emulsion GLA-SE [201,202,203,204]. The recent results demonstrated that GLA-SE significantly improved the immunogenicity of a plant-derived H5 virus-like particle (VLP) vaccine, eliciting protective hemagglutination inhibition (HI) titers in healthy adults [205]. Furthermore, this formulation produced powerful, polyfunctional, and sustained heterologous CD4+ T cell responses targeting the influenza H2 protein, adding to its broad protective potential [206]. Similarly, in murine models, GLA-AF significantly improved the immunogenicity of recombinant H5N1 hemagglutinin protein (rH5) vaccinations based on the clade 1 strain, eliciting HI titers protective against both clade 1 and clade 2 viruses, and imparting considerable protection against heterologous clade 2 viral challenges [207]. While most adjuvants are approved for intramuscular (IM) administration, GLA-AF is evaluated as an intradermal (ID) adjuvant. In murine models that GLA-AF significantly increased HI titers when combined with plant-derived H5 virus-like particle (VLP) vaccine, resulting in enhanced cross-protection against heterologous H5N1 viruses [206]. Moreover, ferrets receiving a single dose of the ID-H5-VLP vaccine with GLA-AF were fully protected against lethal clade 1 challenges, whereas IM administration or ID vaccination without adjuvant induced comparatively weaker responses. This suggests that appropriately activated dermal APCs are more effective in eliciting broad cross-protective immunity than their IM counterparts. Complementary studies indicate that combining TLR4 agonists such as MPL with CpG motifs can potentiate the cross-protective immunological response against heterosubtypic H5N1 viruses in mice [208]. The synthetic combinations of TLR7 and TLR4 agonists, when administered with recombinant or chimeric hemagglutinin (rHA), significantly enhanced cross-protection against diverse influenza strains. Notably, broadly neutralizing antibodies targeting conserved hemagglutinin stalk domains were identified as key mediators for heterosubtypic immunity [209]. Furthermore, heterosubtypic protection was predominantly due to broadly cross-reactive antibodies targeting the hemagglutinin (HA) stalk region.

Overall, TLR4 agonist-based adjuvants (MPL and GLA formulations) robustly enhance vaccine efficacy by promoting broad, durable, and cross-protective immune responses, making them powerful tools for the development of the next generation of universal influenza vaccine.

### 9.8. TLR 7 Adjuvants

Building on the success of TLR4 agonists in enhancing influenza vaccine efficacy, attention turned toward TLR7 agonists for their unique ability to activate antiviral innate immunity. TLR7, a key endosomal sensor of single-stranded RNA viruses like influenza, has been investigated primarily for its adjuvant potential in boosting mucosal and skin-targeted immune responses [210]. Imiquimod, a well-known TLR7 agonist approved for topical treatment of skin lesions, has shown promise as a vaccine adjuvant by enhancing both mucosal and systemic immunity when applied alongside intradermal influenza vaccines. Notably, topical application of imiquimod in combination with intradermal TIV significantly enhanced seroconversion rates in humans, even against drifted influenza strains [211,212]. To enhance local immunogenicity and limit systemic dissemination, structural modifications were introduced to TLR7 agonists, exemplified by the lipid-conjugated 3M-052, which contains an eighteen-carbon alkyl chain to promote retention at the injection site. In murine and ferret models, a single administration of recombinant H5N1 hemagglutinin (rHA) combined with 3M-052 in a stable emulsion conferred complete protection against homologous viral challenge, but rHA alone provided only limited protection [213]. Moreover, the inclusion of 3M-052 in emulsion or liposomal formulations significantly enhanced the generation of neutralizing antibodies against heterologous clade 2 strains. These preclinical findings underscore the potential of TLR7 agonists, such as imiquimod and 3M-052, as effective vaccine adjuvants that amplify both local and systemic immune responses through targeted engagement of innate antiviral pathways, particularly at mucosal and cutaneous sites.

### 9.9. Flagellin

Expanding the repertoire of Toll-like receptor (TLR) agonists explored as vaccine adjuvants, TLR5 agonists such as flagellin offer distinct advantages by directly engaging innate immunity through recognition of bacterial motility proteins. Flagellin, the principal structural protein of bacterial flagella, contains conserved domains that specifically interact with TLR5 and intracellular inflammasomes, enabling potent activation of both humoral and cellular immune pathways [214,215,216]. Structurally, flagellin is composed of four domains: D0, D1, D2, and D3, with highly conserved N- and C-termini forming the D0/D1 domains responsible for interaction with TLR5 and intracellular inflammasomes. In contrast, the D2/D3 domains constitute the highly variable central region. This modular architecture enables flagellin’s unique proteinaceous nature to facilitate genetic fusion with vaccine antigens or co-delivery strategies, streamlining vaccine formulations and potentiating robust immune activation [217]. TLR5 recognizes extracellular flagellin, triggering innate immunological signaling at the cell surface; intracellular flagellin is identified via the cytosolic NLRC4 inflammasome, a member of the NLR family containing a caspase activation and recruitment domain (CARD) domain. Notably, the conserved D0 domain of flagellin plays a crucial role in activating the NLRC4 inflammasome, linking extracellular and intracellular pathogen-sensing pathways to mount a comprehensive immune response [218]. The D1 domain of flagellin is critical for binding to TLR5, facilitating receptor activation. Importantly, the highly variable D3 domain and the N- or C-termini can be modified to incorporate foreign antigens without compromising flagellin’s ability to stimulate TLR5. This structural flexibility has propelled extensive research into flagellin as a versatile and highly immunogenic carrier for vaccines targeting cancer, viruses, bacteria, and parasites. The designed virus-like particles displaying both PR8 hemagglutinin and M1 matrix protein (HA/M1 VLPs), which were further fused with modified flagellin as a molecular adjuvant (flagellin/HA/M1-VLPs). They showed that intranasal vaccination using this formulation provided strong defense against challenges from heterosubtypic H3N2 virus in the murine models. Moreover, flagellin’s capacity to act as a mucosal adjuvant is supported by its potent activation of respiratory epithelial cells expressing TLR5, which induces cytokine and chemokine production [219]. The critical role of co-delivering flagellin alongside HA/M1 virus-like particles in eliciting broad heterosubtypic immunity has been demonstrated by these studies. In summary, flagellin, as a TLR5 agonist, represents a highly promising bio-adjuvant platform that simultaneously engages innate immune pathways while serving as an effective antigen carrier, thereby streamlining vaccine design. Its intrinsic self-adjuvanting properties enable robust immunogenicity without the need for additional adjuvants. Although flagellin-based vaccines have yet to be licensed for influenza, they have advanced through Phase II clinical evaluations, underscoring their potential to significantly enhance future influenza vaccine formulations by eliciting broad, durable, and cross-protective immune responses.

### 9.10. Saponins

Following the exploration of TLR-based adjuvants, saponins have emerged as another class of potent immunostimulatory compounds with significant vaccine adjuvant potential. These are naturally derived glycosides, primarily obtained from the bark (Southern American Tree-Quillaja Saponaria Molina) commonly known as the Chilean soapbark tree, have been extensively studied for their potent immunomodulatory properties. Among them, QS-21 is a highly purified saponin fraction derived from the *Quillaja Saponaria* bark extract, which has been extensively studied for its ability to enhance both humoral and cellular immune responses, making it an important component in modern vaccine formulations [220,221]. QS-21 exhibits potent adjuvant activity when administered alone at high doses; however, its clinical application is limited by dose-dependent hemolytic toxicity [222]. To enhance safety while maintaining efficacy, the QS-21 has been combined with MPL in several adjuvanted formulations. Saponins also form the foundation of immune-stimulating complexes (ISCOMs), particulate assemblies composed of Phospholipids, vaccine antigens, cholesterol, and saponins, which improve antigen delivery and immune activation. More recently, novel saponin-based nanoparticles such as Matrix-M^TM^, incorporating purified *Quillaja saponaria* saponins, have been developed to further refine the balance between safety and immunogenicity in next-generation vaccine adjuvants [223]. Building on their immunostimulatory properties, saponin-containing ISCOMs have been shown to enhance both humoral and cellular immune responses. The application of ISCOMs in influenza vaccination has demonstrated significant potential for inducing cross-protective immunity. For instance, the ISCOMs substantially increased hemagglutination inhibition (HI) titers induced by H1N1 vaccines and provided complete protection in mice against Heterosubtypic H2N2 Viral challenges [224]. Additional research. Further studies established a correlation between cytotoxic T lymphocyte (CTL) response targeting a conserved major histocompatibility complex-I(MHC-I) epitope within the hemagglutinin (HA) proteins of H1 and H2 influenza viruses and the heterosubtypic protection conferred by ISCOMs [25,225].

Extending the promising findings with ISCOMs, newer saponin-based adjuvants such as Matrix-M^TM^, developed by Novavax, have demonstrated substantial efficacy in influenza vaccine research. When formulated with a virosomal vaccine, Matrix-M^TM^ significantly enhanced cross-protective immunity against avian influenza strains H5 and H7 in animal models. Similarly, the incorporation of Matrix-M^TM^ into trivalent virosomal vaccines markedly increased hemagglutination inhibition (HI) titers and protection against heterologous influenza B viruses, although such enhancement was not observed for influenza A viruses. Additionally, Matrix-M^TM^ substantially amplified HI titers induced by hemagglutinin (HA) nanoparticles and improved protection in ferrets against antigen-drifted H3N2 virus strains, underscoring its potential as a powerful adjuvant platform for broad influenza vaccine efficacy [226]. The Matrix-M^TM^ adjuvanted trivalent virosomal vaccine produced strong cross-protective immunity against highly pathogenic H5N1 and H7N7 influenza viruses in both mouse and ferret models, further supports these findings and highlights the adjuvant’s potential to improve broad-spectrum influenza vaccine responses [227].

The promising preclinical and early clinical findings on saponin-based adjuvants extended into Phase 1 trials, where Matrix-M™, combined with a virosomal H5N1 influenza vaccine, produced a robust antibody response against both heterologous and homologous H5N1 strains. Building on these encouraging results, Matrix-M™ advanced to Phase 3 randomized controlled trials as part of a quadrivalent nanoparticle influenza vaccine formulation, further validating its potential to enhance both pandemic and seasonal influenza vaccine efficacy through broad immunogenic coverage [227,228]. Following the H7N9 avian influenza virus’s appearance in China in October 2013, Novavax Inc. announced the successful completion of its clinical trials for an H7N9 vaccine candidate using Matrix-M™ as an adjuvant, underscoring the accelerating pace of adjuvanted influenza vaccine development to address emerging pandemic threats [229,230]. Researchers used a baculovirus expression system containing the M1 protein from A/Indonesia/5/2005 (H5N1), together with the HA and NA proteins from A/Anhui/1/2013 (H7N9) evaluate the immunogenicity of an H7N9 virus-like particle (VLP) vaccine formulated with a saponin-based immunostimulatory complexes and matrix (ISCOMATRIX) adjuvant, which is made of a nanoparticle formulation containing saponin, cholesterol, and phospholipids. Despite the inherently lower immunogenicity associated with the H7 hemagglutinin, the inclusion of the saponin-based adjuvant elicited superior hemagglutination inhibition (HI) responses compared to formulations with alum or without any adjuvant [231]. These findings align with observations from seasonal influenza vaccines, demonstrating that saponin-based adjuvants consistently enhance immune responses even against less immunogenic influenza strains when appropriately paired with the vaccine antigen.

In comparative evaluations against licensed quadrivalent inactivated influenza vaccines, Matrix-M™ adjuvanted formulations demonstrated enhanced humoral and cellular immune responses in individuals aged 65 years and older, a demographic with typically weaker vaccine responsiveness. Matrix-M™ facilitated substantial antigen dose sparing while maintaining high immunogenicity, highlighting its strategic value in pandemic and routine immunization programs. Importantly, Matrix-M™ also facilitated significant antigen dose sparing while sustaining high immunogenicity, emphasizing its strategic value in both pandemic settings and routine vaccination programs [232]. Given their proven efficacy in licensed non-influenza vaccines, saponin-based adjuvants like Matrix-M™ hold considerable promise for broadening and strengthening protective responses in seasonal and universal influenza vaccine platforms.

### 9.11. Enterotoxin Adjuvants

With the proven efficacy of systemic adjuvants in injectable influenza vaccines, research has increasingly shifted toward identifying adjuvants that can effectively enhance mucosal immunization. Bacterial enterotoxins, such as cholera toxin (CT) from Vibrio cholerae and heat-labile enterotoxin (LT) from enterotoxigenic E. coli, have been extensively studied for their capacity to enhance immune responses during intranasal and oral influenza vaccination [233].

CT and LT, members of the AB5 toxin family, comprise one enzymatically active A subunit responsible for cellular toxicity and five B subunits that mediate binding to the Ganglioside receptors (GM-1) on target cells via a pentameric structure. Intranasal administration of cholera toxin (CT) significantly enhanced the hemagglutination inhibition (HI) titers elicited by whole-inactivated PR8 (PR8i) vaccine in mice, conferring robust protection against heterologous and heterosubtypic influenza virus challenges. Notably, PR8i alone induced only modest defense against Heterosubtypic H3N2 viruses, whereas the CT-adjuvanted formulation provided complete protection, attributed primarily to cross-reactive antibodies rather than T cell responses [234]. Complementing these findings, the CTB (cholera toxin B subunit) markedly improved intranasal TIV-induced cross-protection against drifted influenza strains in mice, an effect was absent when the vaccine was administered subcutaneously, highlighting the critical role of mucosal delivery in achieving broad-spectrum immunity [235] Clinically, this strategy was exemplified by Nasalflu, an intranasal influenza vaccine licensed in Switzerland in 2000, which combined inactivated influenza antigens with an LT derivative; although its approval was short-lived, it highlighted the translational potential of enterotoxin-based mucosal adjuvants. Despite the initial success of Nasalflu, its association with an increased risk of Bell’s palsy led to its market withdrawal, underscoring the safety concerns surrounding enterotoxin-based adjuvants in humans. Attenuated derivatives of LT, the single-mutant LT (R192G) and the double-mutant LT (R192G/L211A), were developed to minimize enterotoxin effects while retaining immunostimulatory properties to address toxicity concerns [236]. In this context, the intranasal administration of H3N2 vaccine alone conferred only limited protection against highly pathogenic H5N1 challenge in mice, co-administration with LT (R192G) mutant adjuvant achieved complete protection, reinforcing the potential of detoxified enterotoxin variants as mucosal adjuvants for enhancing cross-protective immunity [25,237].

Moreover, subcutaneous immunization with LT or Freund’s adjuvant did not protect against lethal H5N1 challenges, underscoring the essential role of mucosal administration. Notably, the B-cells rather than CD8+ T-cells were found to be the primary effectors driving heterosubtypic immunity in this setting. Enterotoxin-based adjuvants, when carefully attenuated, continue to represent some of the most potent mucosal adjuvants, capable of inducing both secretory IgA and cytotoxic T cell responses within the respiratory tract. With ongoing advancements in safety modifications, these adjuvants hold promise for enabling intranasal vaccines that establish robust, localized immunity at the primary sites of influenza infection, the nasal mucosa and lungs, thereby providing broad cross-protective efficacy. For instance, prior studies demonstrated that intranasal administration of an inactivated influenza vaccine adjuvanted with LT mutants successfully protected mice against lethal H5N1 challenge via the induction of heterosubtypic immunity [237]. Collectively, these adjuvant strategies represent the forefront of influenza vaccine development, poised to enable more effective and broadly protective vaccines for the future.

### 9.12. Physical Adjuvants

Physical adjuvants have been explored in parallel as innovative alternatives to chemical agents, with the goal of boosting immune responses without the use of biological components. By utilizing physical energies such as radiofrequency and lasers, these adjuvants promote skin irritation and activate innate immune pathways to boost intradermal and transdermal vaccine delivery. Their transient, minimally invasive application reduces the risk of long-term side effects, and preclinical studies have demonstrated the effectiveness of laser adjuvants in improving vaccines for influenza, nicotine addiction, and cancer [238]. Recent clinical testing of a near-infrared laser adjuvant revealed that it significantly increased cutaneous immune cell trafficking in people while maintaining high safety and tolerability. Similarly, in murine models, it has been revealed that non-ablative fractional laser (NAFL) can safely boost ID influenza vaccination by purposefully producing microscopic photothermal damage [239]. Building on this, mouse model studies revealed that NAFL substantially enhanced cross-protection against heterologous H1N1 and H3N2 influenza strains, as well as immune responses triggered by dissolving microneedle-based PR8i vaccination. Similarly, radiofrequency adjuvant (RFA) was shown to simultaneously boost both humoral and cellular immunity in intradermal influenza vaccination, with efficacy at a 0.3 µg dose comparable to the MF59-like AddaVax adjuvant and surpassing AddaVax at a lower 0.06 µg dose [240].

Following the advances in mucosal, chemical adjuvants, and physical adjuvants offer a novel approach by enhancing vaccine delivery and mimicking natural danger signals to boost immune activation. Emerging technologies, like self-applied microneedle patch flu vaccines, can mechanically amplify immune responses. These methods are especially promising for achieving broad cross-protection by promoting robust T-cell responses and mucosal IgA, critical for heterosubtypic influenza immunity.

## 10. Adverse Effects Related to Adjuvanted Vaccines

While adjuvants significantly enhance vaccine effectiveness, their inclusion also requires careful evaluation of safety and reactogenicity. Influenza vaccines, including adjuvanted formulations, have an outstanding safety record, with billions of doses administered globally and few serious adverse events reported. Nevertheless, adjuvants can increase the frequency of mild-to-moderate local and systemic reactions, and rare immune-mediated events have been observed. Thus, balancing the clear immunological benefits of adjuvants with vigilant safety monitoring remains crucial to ensure optimal protection with minimal risk. Water-in-oil (W/O) emulsions, such as Freund’s adjuvant, are powerful immunostimulants that can produce strong and lasting immune responses, making them effective in preclinical and veterinary settings. Despite their efficacy, their use in human medicine is severely limited due to significant safety concerns [241,242]. These adjuvants can cause a range of severe local reactions at the injection site, including intense pain, inflammation, abscesses, and ulceration [243]. Due to reports of both local and systemic side effects, questions have also been raised about the safety of the oil-in-water adjuvant AS03, which is used in the pandemic H1N1 influenza vaccination. Although these reactions were generally mild, an increased incidence of narcolepsy has also been observed in children vaccinated with AS03-adjuvanted formulations in certain countries. Current evidence suggests that the pandemic H1N1 viral proteins, rather than the AS03 adjuvant itself, may trigger narcolepsy. This condition, narcolepsy type 1, arises from autoimmune destruction of hypocretin-producing neurons [244,245]. The pandemic H1N1 influenza virus’s viral antigen or antigens may have triggered localized CD4+ T cell responses that cross-react with hypocretin peptides, due to molecular resemblance between viral peptides and hypocretin, leading to immune-mediated damage of hypocretin-producing neurons.

Clinical studies comparing Alum and MF59 adjuvants in recombinant split H5N1 influenza vaccines demonstrated that MF59 significantly enhanced neutralizing antibody responses compared to alum, without causing major adverse effects [162,246]. Systemic reactions such as fever, malaise, and headaches were similar in both groups, though 70% of individuals receiving MF59 reported mild to moderate local discomfort and tenderness at the injection site. While no definitive causal relationship between adjuvants and serious adverse events has been established, ongoing vigilance and investigation remain essential to further improve vaccine safety and public confidence [81].

The safety profile of influenza vaccines is influenced by multiple factors, particularly the route of administration and the choice of adjuvant strategies. The intramuscular (IM) route is generally preferred for vaccines containing adjuvants, as alternative routes such as subcutaneous or intradermal injection are more frequently associated with local adverse reactions, including irritation, inflammation, skin discoloration, and in some cases, granuloma formation [247]. To ensure continued safety following widespread vaccine deployment, robust pharmacovigilance systems are essential for detecting adverse events at an early stage [248]. Passive surveillance programs, such as the Vaccine Adverse Event Reporting System (VAERS) in the U.S. and equivalent systems in other regions, serve as critical early warning tools. These systems facilitate the timely identification of rare or unexpected side effects, which can then be further investigated to maintain public confidence in vaccine safety [249].

## 11. Universal Influenza Vaccine (UIV)

Addressing the challenges posed by current seasonal vaccines and the evolving nature of influenza viruses, the pursuit of a universal influenza vaccine has gained urgency. Unlike traditional seasonal vaccines, which target specific and often rapidly evolving viral strains, a universal vaccine seeks to generate robust immune responses against conserved viral components shared across diverse influenza subtypes [250]. Here are a few approaches that may facilitate the development of broad-spectrum influenza vaccines:

### 11.1. Targeting Viral Proteins

The limitations of current influenza vaccines, which primarily target the highly variable “head” of the HA protein, underscore the need for a new generation of universal vaccines. These next-generation vaccines are designed to provide broad, continuing protection against a wide range of influenza A and B viruses, including those with pandemic potential. This is achieved by shifting the focus from the mutable HA head to more conserved viral proteins that are essential for the virus’s life cycle and are less prone to mutation [251]. Among the most promising of these targets are the matrix protein-M2 and nucleoprotein (NP), which have been shown the cross-protective and robust immune response. Studies in animals show that NP and M2 vaccines elicit broad, short-term protection against influenza A viruses, including avian H5N1. Mucosal vaccination is superior for boosting, and a single intranasal dose provides extended protection. This suggests that targeting these conserved viral proteins via mucosal delivery is a highly effective strategy for developing a universal influenza vaccine [252].

### 11.2. T-Cell-Based Vaccines

Influenza-specific T cells constitute an essential element in the formulation of universal vaccines. T-cell-based strategies aim to elicit robust cytotoxic T lymphocyte (CTL) responses against conserved internal viral proteins, such as NP and matrix protein-1 (M1), which can provide cross-protection across diverse influenza strains [253]. Despite progress, key obstacles include the short lifespan of tissue-resident memory T cells, viral immune escape mechanisms, and the influence of antigen presentation on immunodominance hierarchies across different Human Leukocyte Antigen (HLA) types [254]. Innovative platforms, including DNA and virus-based vaccines incorporating these conserved proteins, have demonstrated the ability to generate potent T-cell immunity, enhancing the breadth of immune defense beyond antibodies [255]. Further research is needed to define the protective capacity of T cells and optimize vaccine designs that safely elicit durable memory populations. Modeling studies suggest that T cell-targeted vaccines may be particularly effective in antibody-naïve, T cell-primed individuals, such as in the context of emerging strains like H7N9, compared with seasonal viruses like H3N2 [256].

### 11.3. mRNA-Based Vaccine

mRNA influenza vaccines have emerged as a transformative platform by targeting conserved viral components such as ectodomain of the M2 ion channel (M2e), long alpha helix of haemagglutinin stalk region (LAH), and NP, thereby eliciting strong antibody and T-cell responses capable of protecting against diverse influenza subtypes (H1N1, H3N2, H9N2) [257]. The first clinical studies using LNPs (Lipid Nanoparticles) chemically modified mRNA vaccines against H10N8 and H7N9 influenza viruses demonstrated favorable safety and immunogenicity profiles, and serious adverse events associated with the vaccine were not reported. Seroconversion rates reached 78.3% hemagglutination inhibition (HAI) and 87.0% microneutralization (MN) for H10N8, while responses for H7N9 were even higher at 96.3% (HAI) and 100% (MN). Building on these promising outcomes, next-generation approaches have broadened the design of mRNA vaccines. For example, in mice and ferrets, nucleoside-modified mRNA–LNP vaccines expressing HA antigens from all 20 influenza A and B subtypes induced robust cross-reactive and subtype-specific antibody responses, protecting against related and distant strains [258]. Similarly, stalk-targeted mRNA vaccines have demonstrated the capacity to elicit broadly reactive antibody responses, protection against homologous, heterologous, and heterosubtypic influenza viruses. Notably, candidates such as H1ssF-3928 mRNA-LNP are currently undergoing phase 1 clinical evaluation. This innovative approach offers the potential for a single, broadly protective vaccine with durable immunity [259]. Additionally, mRNA platforms inherently stimulate innate immunity, acting as self-adjuvants, and their flexibility allows for rapid updates, positioning them as a leading strategy for future influenza vaccine development, accelerated by the success of COVID-19 mRNA vaccines [257].

### 11.4. Epitope-Based Vaccine Design

Harnessing advanced bioinformatics and immunological data, researchers are strategically selecting conserved epitopes from multiple influenza proteins to create a unified, highly immunogenic vaccine candidate. This precision-designed approach targets broad-spectrum immunity by combining antigenic fragments from diverse viral strains, effectively anticipating viral evolution and mutation [260]. Nucleoprotein (NP) and matrix protein 1 (M1), internal components of influenza viruses, exhibit high conservation and contain numerous T cell epitopes, making them attractive targets for T-cell-based vaccines. NP is conserved across influenza A virus strains and has been extensively studied for its role in eliciting protective T cell immunity. The nucleoprotein and M1-targeted vaccines stimulate CD4+ and CD8+ T cell responses, providing broad-spectrum immunity that can mitigate disease severity and duration. The MVA-NP+M1 vaccine, a modified vaccinia Ankara vector expressing NP and M1, showed good tolerability in Phase 1 trials, although Phase IIb studies failed to reach their primary endpoints [261,262]. These findings highlight both the promise and challenges of T-cell-targeted strategies, underscoring the need for continued optimization to achieve robust and durable protection [263,264].

### 11.5. Chimeric Hemagglutinin (cHA)

Chimeric hemagglutinin (cHA)-based vaccines represent a promising strategy to elicit antibodies against the immunosubdominant HA stalk. By combining exotic avian HA head domains, such as H8 and H5, with a conserved seasonal HA stalk, cHAs can selectively boost anti-stalk antibodies, which are further amplified upon sequential vaccination with constructs carrying different heads but the same stalk [265,266]. A recent phase 1 clinical trial in adults (18–39 years) demonstrated that the adjuvanted, inactivated cHA vaccine produces high and long-lasting anti-stalk antibody titers with functional Fc-mediated activity, even after a single dose, while all regimens were well tolerated. Together with preclinical studies in multiple animal models, these results underscore the potential of cHAs to elicit broad and durable protection [267,268]. The development of stalk and Influenza B Virus (IBV) mosaic HA constructs could lead to a trivalent universal influenza vaccine offering broad protection against diverse influenza strains [269].

## 12. Conclusions

Despite decades of vaccine development, influenza virus persists as a major global health threat, reinforcing the urgent demand for vaccines that confer broad, durable, and cross-strain immune protection. Incorporating adjuvants into vaccine formulations remains one of the most promising strategies to achieve this goal. Classical adjuvants, with their well-established safety profiles and ease of production, provide a strong foundation for vaccine development, yet their limited ability to stimulate broad immune responses underscores the necessity for innovation. Emerging adjuvants, targeting specific immune components, have demonstrated the capacity to enhance cross-protective immunity, enable antigen dose-sparing, and expand immune recognition. In addition, new delivery methods such as nanoparticle-based carriers, liposomes, and polymeric systems are being developed to improve antigen stability and presentation. Alternative administration routes, including intranasal, transdermal, and mucosal delivery, are also being explored to more effectively target immune cells at their natural sites of activation. The new delivery systems also repurpose the traditional adjuvants through surface modification, formulation refinement, and combination strategies, offering a clinically relevant path forward. Ultimately, the rational design of next-generation adjuvants, guided by advances in immunological insights, will be central to developing influenza vaccines that are not only highly immunogenic, but also safe, scalable, and broadly effective across diverse population, which we attempted to summarize in this review. 

## Figures and Tables

**Table 1:** The FDA approved influenza seasonal vaccines. Information adopted from the U.S. Food and Drug Administration, vaccine licensed for use in the United States; content is current as of 31 May 2025 (https://www.fda.gov/vaccines-blood-biologics/vaccines/vaccines-licensed-use-united-states) accessed on 23 August 2025.

Vaccine Type	Trade Name	Adjuvant	Production Platform	How Supplied	Route	Dose	Age Range	Manufacturer
Subunit	FLUAD	MF59	Embryonated egg	0.5 mL (single-dose syringe)	i.m	45 µg (15 µg HA/strain)	≥65 yrs	CSL Seqirus
	FLUAD (Quadrivalent)	MF59	Embryonated egg	0.5 mL (single-dose syringe)	i.m	60 µg (15 µg HA/strain)	≥65 yrs	CSL Seqirus
	Flucelvax (Quadrivalent) (Cell culture)	None	Embryonated egg	0.5 mL (single-dose syringe)	i.m.	45 µg (15 µg HA/strain)	6 months & older	CSL Seqirus
Live attenuated	FluMist	None	Embryonated egg	0.2 mL (single-use)	i.n	10^6.5–7.5^ fluorescent focus units of each vaccine virus	2 to 49 yrs	AstraZeneca
Recombinant hemagglutinin influenza vaccine	Flublok	None	Baculovirus/Insect cell	0.5 mL (single-dose syringe)	i.m	135 µg (45 µg HA/strain)	≥18 yrs	Sanofi
	FluBlok Quadrivalent	None	Baculovirus/Insect cell	0.5 mL (single-dose syringe)	i.m	180 µg (45 µg HA/strain)	≥18 yrs	Sanofi
Split	Afluria	None	Embryonated egg	5.0 mL multi-dose vial (0.25 mL dose)	i.m	45 µg (15 µg HA/strain)	6–35 months	CSL Seqirus
	Afluria	None	Embryonated egg	5.0 mL multi-dose vial (0.5 mL dose)	i.m	45 µg (15 µg HA/strain)	3 yrs & older	CSL Seqirus
	Fluarix	None	Embryonated egg	0.5 mL (single-dose)	i.m	45 µg (15 µg HA/strain)	6 months & older	GSK
	FluLaval	None	Embryonated egg	0.5 mL (single-dose syringe)	i.m	45 µg (15 µg HA/strain)	≥6 months	GSK
	FluLaval Quadrivalent	None	Embryonated egg	0.5 mL (single-dose)	i.m	45 µg (15 µg HA/strain)	≥6 months	GSK
	Fluzone Quadrivalent	None	Embryonated egg	0.5 mL (single-dose)	i.m	60 µg (15 µg HA/strain)	≥6 months	Sanofi
	Fluzone High-Dose Quadrivalent	None	Embryonated egg	0.5 mL (single-dose)	i.m	180 µg (60 µg HA/strain)	65 yrs & older	Sanofi

i.n: intranasal spray, i.m: intramuscular injection, GSK: GlaxoSmithKline.
